# Age-Related Olfactory and Cognitive Decline: Potential Effects of *Rosmarinus officinalis* and *Carum carvi* Essential Oils

**DOI:** 10.3390/nu18050862

**Published:** 2026-03-07

**Authors:** Antonella Rosa, Alessandra Piras, Silva Porcedda, Paolo Solari, Ilenia Pinna, Carla Masala

**Affiliations:** 1 Department of Biomedical Sciences, University of Cagliari, SP 8 Cittadella Universitaria, 09042 Monserrato, CA, Italy; anrosa@unica.it (A.R.); solari@unica.it (P.S.); ilenia.pinna@unicam.it (I.P.); 2 Department of Chemical and Geological Sciences, University of Cagliari, SP 8 Cittadella Universitaria, 09042 Monserrato, CA, Italy; apiras@unica.it (A.P.); porcedda@unica.it (S.P.)

**Keywords:** essential oils, *Rosmarinus officinalis*, *Carum carvi*, flavor, aging, olfaction, cognitive abilities, carvone, limonene, 1,8-cineole

## Abstract

Background: Aging is characterized by a decrease in olfactory, attentional, memory, language, and visuospatial/executive abilities. In this context, our study aimed to evaluate the potential effects of *Rosmarinus officinalis* L. (rosemary) and *Carum carvi* L. (caraway) essential oils (EOs) on aging. First, we assessed, in 402 participants, the age-related changes in olfactory functions (odor threshold, discrimination, and identification), gustatory perceptions (sweet, sour, salty, and bitter taste), cognitive functions (focusing on attention, memory, language, and visuospatial/executive functions), and their possible correlations with aging. To achieve this, olfactory function, gustatory perception, and cognitive abilities were evaluated in healthy participants across different age groups. Then, to evaluate the age-related decrease in trigeminal function (59 participants), we used rosemary and caraway EOs that contain carvone, limonene, and 1,8-cineole, all of which are considered typical trigeminal stimuli. Methods: Olfactory function was assessed with the Sniffin’ Sticks test, gustatory function by the Taste Strips test, and rosemary and caraway EOs by the ratings of odor pleasantness, intensity, and familiarity using a labeled hedonic Likert-type scale. Results: Olfactory function could be a potential early indicator of attentional, memory, language, and visuospatial/executive dysfunctions. Our data indicated that rosemary and caraway EOs were perceived without any significant decrease in odor pleasantness, intensity, and familiarity ratings in relation to aging. Conclusion: Our results suggest the potential bioactive effects of rosemary and caraway natural EOs as a new strategy to promote healthy aging.

## 1. Introduction

Aging is considered a biological, progressive process associated with cellular changes and both anatomical and physiological modifications in the human body. These modifications may induce a physiological decline in different functions such as the cardiovascular and musculoskeletal systems, the brain, and the sensory system [1]. The age-related decline has been recently associated with an increase in reactive oxygen species (ROS), leading to oxidative stress, protein oxidation, DNA damage, lipid peroxidation, and mitochondrial dysfunction [2].

In the sensory system, the main age-related alterations are olfactory, gustatory, trigeminal, visual, and auditory dysfunctions [3,4,5]. Olfactory, gustatory, and trigeminal functions are important components of flavor perception, which regulate food preference and palatability [6]. The olfactory system is particularly vulnerable to the effects of aging because it is continuously exposed to toxic environmental substances such as ionized metals (cadmium, aluminum, and manganese), which may enter the brain through the olfactory nerve [7]. Several studies have documented decreases in olfactory receptor numbers and the volume of the olfactory bulb with age [8,9]. Some previous studies have indicated a decrease in trigeminal sensitivity in elderly subjects [10,11]. The age-related decrease in the trigeminal chemosensory system is associated with a decline in tactile, thermal, and chemical sensitivity in the nasal epithelium [6]. The intranasal trigeminal sensation is a protective mechanism mediated by two types of fibers, i.e., the *C*-fibers and the A_delta_-fibers. *C*-fibers are involved in burning sensations, while A_delta_-fibers are involved in stinging sensations [12,13]. Trigeminal sensations, such as coolness, burning, and stinging, can also be perceived in patients with olfactory deficits [13,14]. Consequently, the interaction between the olfactory and trigeminal systems is a complex pathway that may be difficult to predict with respect to age, but it strongly influences odor perception [11]. Compared with the olfactory system, the trigeminal system has received less attention over the years. Most odorants may stimulate the trigeminal system in addition to the olfactory system, at least at higher concentrations; therefore, the olfactory and trigeminal systems work closely together in the perception of an olfactory stimulus [13,14]. Humans are able to perceive mixed olfactory/trigeminal stimuli, and natural volatile compounds such as eucalyptol may activate both the olfactory and the trigeminal nerves [14]. It has been demonstrated that the stimulation of the trigeminal nerve can improve olfactory function in individuals with hyposmia, potentially enhancing the ability to perceive olfactory information [15].

Olfactory deficits often precede cognitive decline, such as decreased verbal fluency, memory, attention, and visuospatial abilities [16]. An olfactory deficit is often considered a marker of cognitive decline, as olfactory functions have been associated with changes in the volume and metabolism of the medial temporal cortex [17,18,19]. Olfactory dysfunction has been reported in various conditions, including Alzheimer’s disease, Parkinson’s disease, Huntington’s disease, and amyotrophic lateral sclerosis [20]. The mechanism of cognitive dysfunction could be induced by oxidative stress and chronic neuroinflammation [21]. In neuronal structures, the main age-related decline involves synaptic dysfunction, characterized by a reduction in the number and length of dendritic spines, astrocyte hypertrophy, neuroinflammation, reduced volume of brain regions (such as the olfactory bulb, piriform cortex, and amygdala), and decreased myelination [8,22]. The age-related decline in neuronal structure is associated with a reduced quality of life, characterized by difficulties in correctly processing sensory information from the environment, malnutrition, changes in body weight, and an increased risk of chronic diseases [9].

Over the past 30 years, there has been a dramatic increase in cognitive dysfunction and dementia in the global population. Therefore, early detection of olfactory and trigeminal function is important to delay or prevent cognitive impairment, together with the identification of innovative strategies to address age-related olfactory deficits and cognitive disorders [23].

Natural essential oils (EOs) are complex liquid mixtures of volatile, aromatic, and lipophilic compounds, synthesized as secondary metabolites by plants [24,25,26]. EOs are often used for their biological activities, including antioxidant, anti-inflammatory, antimicrobial, antiviral, analgesic, and anticancer properties [24,25,26]. The inhalation of EOs has been demonstrated to improve the sense of smell in patients with olfactory deficits [26]. An increasing number of studies have suggested the role of EO inhalation in the activation of the central nervous system and improvement of cognitive function, through the stimulation of neural pathways involved in emotion and memory [27,28,29]. EOs have been shown to reduce neurotoxicity, neuroinflammation, and oxidative stress during aging [27]. The main components of EOs, such as terpenes, terpenoids, and polyphenols, are small, soluble organic molecules and are absorbed through the respiratory tract or skin, can enter the blood circulation, may cross the blood–brain barrier, and produce systemic effects [21,26,27,29]. The olfactory pathway involves a direct route from olfactory receptors in the nasal cavity to the olfactory bulb that has anatomical connections with the amygdala and hippocampus, important in emotion and memory, respectively [21]. EOs’ effects may be exerted by directly increasing the amount of acetylcholine that persists in the synaptic space and could interact with postsynaptic receptors [21]. Cardamom EO, rich in 1,8-cineole, has previously been linked to cognitive enhancement in healthy adults [28]. Moreover, lemongrass EO has been shown to improve attention and memory [28]. Lemon, bergamot, and lavender EOs have demonstrated anti-dementia effects, reducing the neurological dysfunction [25]. Interestingly, many natural EO components are able to stimulate trigeminal sensation, including carvacrol (from oregano), eugenol (cloves), and thymol (thyme), among others [30].

Among aromatic plants, *Rosmarinus officinalis* L. (rosemary) and *Carum carvi* L. (caraway) are perennial herbs widely distributed around the world. Rosemary is an aromatic plant used as a flavoring agent in food in the Mediterranean area. The presence of polyphenols and tannins in the rosemary composition suggested an antioxidant activity of the plant [31]. Rosemary EO showed different bioactive properties such as antioxidant, antiviral, antibacterial, antiseptic, and antitumor effects [31,32,33,34,35]. Previous studies indicated that rosemary EO may improve memory, attention, cognitive function, and mood by direct stimulation of acetylcholine, an important neurotransmitter involved in learning and memory processes [35]. Inhalation of rosemary EO allows volatile compounds to enter the bloodstream via the respiratory system, leading to increased blood levels of 1,8-cineole, one of the main components of rosemary EO. 1,8-Cineole is considered a typical trigeminal stimulant in humans with a cooling effect [36].

An aromatic plant that is less well-known than rosemary is caraway, traditionally used as a spice in foods and beverages in Europe, Tunisia, Iran, and Egypt. Caraway EO showed bioactive properties such as expectorant, carminative, anti-hyperglycemic, anti-hyperlipidemic, antispasmodic, anti-inflammatory, and antiulcerogenic effects [37,38]. The caraway EO chemical analyses showed a high concentration of carvone and limonene [38]. Also, carvone and limonene are typical human trigeminal stimulants [36].

To the best of our knowledge, no previous studies have properly evaluated the potential effects of rosemary and caraway EOs on the olfactory, gustatory, and trigeminal functions in relation to age. In this context, our cross-sectional study aimed to evaluate the potential effects of rosemary and caraway EOs on improving the age-related chemosensory decline. First, we assessed the age-related changes in olfactory functions, gustatory perceptions (sweet, sour, salty, and bitter tastes), and cognitive functions (focusing on attention, memory, language, and visuospatial/executive functions), and their possible correlations with aging. To achieve this, olfactory function, gustatory perception, and cognitive abilities were evaluated in healthy participants across different age groups. Then, differences in the odor ratings of pleasantness, intensity, and familiarity of rosemary and caraway EOs were evaluated in a subpopulation of young adults and middle-aged participants to assess their effects on the chemosensory system in relation to age and explore the potential role of trigeminal stimulation in improving the age-related chemosensory decline.

## 2. Materials and Methods

### 2.1. Essential Oils

The tested *C. carvi* EO was obtained by supercritical extraction from the seeds of the plant harvested in Lithuania, as previously described [38], while the *R. officinalis* EO was supplied by the company Erbe Matte, lot no. 03/2015 (Sant’Antioco, SU, Italy).

### 2.2. GC-MS Analysis of Essential Oils

EO analysis was carried out by gas chromatography/mass spectrometry (GC-MS), using a gas chromatograph (Agilent 7820A, Agilent Technologies, Santa Clara, CA, USA) equipped with a 30 m × 0.25 mm i.d. with 0.25 µm stationary film thickness DB-5MS UI capillary column (Agilent J&W, Agilent Technologies, Santa Clara, CA, USA), coupled with a mass selective detector having an electron ionization device (EI) and a quadrupole analyzer (Agilent 5975 MSD, Agilent Technologies, Santa Clara, CA, USA), as previously reported [26]. The following temperature program was used: from 60 °C to 246 °C at a rate of 3 °C min^−1^ and then held at 246 °C for 20 min (total analysis time 82 min). Other operating conditions were the following: carrier gas helium (purity ≥ 99.9999%, Air Liquide Italy, Milano, Italy); flow rate, 1.0 mL/min; injector temperature, 250 °C; detector temperature, 300 °C. Injection of 1 μL of diluted sample (1:100 in *n*-hexane, *w*/*w*) was performed with 1:20 split ratio, using an autosampler (Agilent, Model 7683, Agilent Technologies). The conditions used for MS analysis were as follows: MS transfer line temperature, 240 °C; EI ion source temperature, 200 °C with ionization energy of 70 eV; quadrupole temperature, 150 °C; scan rate, 3.2 scan s^−1^ at m/z scan range (30 to 480). The Agilent MSD ChemStation G1701EA software (rev. E.01.00.237, Agilent Technologies, Santa Clara, CA, USA) was used to handle and analyze chromatograms and mass spectra. Compounds were identified by comparing their experimental retention indices and mass spectra with those reported in the literature and library spectra [39,40]. Retention indices of the components were calculated by using the retention times of two standard mixes of *n*-alkanes (C_8_–C_20_ and C_21_–C_40_) [26,41]. The percentage of individual components was calculated based on GC peak areas (semi-quantitative analysis by peak area normalization) as previously reported [26,42].

### 2.3. Participant Enrollment and Study Design

This cross-sectional study, using convenience sampling, enrolled 402 participants aged between 18 and 86 years, divided into three groups: young adults (18–29 years, *n* = 178, 90 women and 88 men), middle-aged (30–59 years, *n* = 137, 63 women and 74 men), and elderly (60 years and older, *n* = 87, 47 women and 40 men). All participants provided informed consent to take part in the evaluation. The assessments were conducted at the University of Cagliari, Italy. The study received approval from the Ethical Committee of the University of Cagliari (protocol number: 3605, 1 October 2024) and was carried out in accordance with the Declaration of Helsinki. For all participants, the following data were collected: weight (kg), age, height (m), body mass index (BMI, kg/m^2^), smoking status, and gustatory and olfactory functions. Inclusion criteria included being 18 years or older, being in overall good health, and being able to understand and perform the test procedure adequately.

In the present study, exclusion criteria were respiratory infections, chronic/acute rhinosinusitis, history of head or neck trauma, neurodegenerative disease, any systemic disease associated with olfactory and gustatory disorders, any cancer or treatment for cancer (chemotherapy), diabetes, pregnancy, severe cognitive decline, and asthma. All participants were assessed individually in a well-ventilated and comfortable room. The complete session test was around 1 h, including olfactory, gustatory, and cognitive functions, and odor rating of the two EOs. Participants were instructed to drink only water 1 h before the experiment and not to wear any scented products on the day of testing. All assessments were carried out at room temperature (23 °C) in a ventilated room during the daytime (9 a.m. to 6 p.m.).

### 2.4. Assessment of Olfactory and Gustatory Functions

The olfactory function was evaluated by the Sniffin’ Sticks test (Burghart Messtechnik, Wedel, Germany) [43,44,45], which consists of three subtasks: odor threshold (OT), odor discrimination (OD), and odor identification (OI). First, OT was assessed using *n*-butanol with 16 stepwise dilutions. The OT was measured using the single-staircase technique based on a three-alternative forced-choice task (3AFC). Second, OD was measured for 16 trials, in each discrimination, three pens were presented, two containing the same odor and the third containing the target odorant (3AFC task). Third, OI was measured using 16 common odors, each presented with four verbal descriptors in a multiple forced-choice format. The interval between odor presentations was 20–30 s. The global olfactory function (TDI score) is calculated by summing the thresholds, discrimination, and identification scores. The scores ≤ 16, between 16.25 and 30.5, between 30.75 and 41.25, and >41.5 were classified as functional anosmia, hyposmia, normosmia, and supersmellers, respectively [46]. The assessment of gustatory function was done by the Taste Strips test (Burghart Messtechnik, Wedel, Germany). This taste test consists of filter paper strips containing four different concentrations of each basic taste quality (sour: 0.3, 0.165, 0.09, and 0.05 g/mL of citric acid; sweet: 0.4, 0.2, 0.1, and 0.05 g/mL of sucrose; bitter: 0.006, 0.0024, 0.0009, and 0.0004 g/mL of quinine hydrochloride; salty 0.25, 0.1, 0.04, and 0.016 g/mL of sodium chloride) [47]. The global gustatory function, which is the sum of all correct values for each taste modality, may vary from 0 to 16 (a score < 9 is classified as hypogeusia) [34].

### 2.5. Assessment of Cognitive Abilities

Cognitive performance in all subjects was assessed using the Montreal Cognitive Assessment (MoCA) Italian version, a rapid screening test for mild cognitive impairment. This test assesses various domains, including attention, memory, language, executive function, and orientation. The maximum potential score is 30, and a score of 26 and above is considered normal [48,49]. In each subject enrolled in the study, and according to the MoCA scale guidelines, one point has been added for participants who have 12 years or fewer of formal education.

### 2.6. Evaluation of Odor Pleasantness, Intensity, and Familiarity of Rosemary and C. carvi EOs

Among all participants, a subpopulation of young adults (18–29 years) and middle-aged (30–59 years) subjects was randomly enrolled to assess odor pleasantness, intensity, and familiarity of rosemary and *C. carvi* EOs. Elderly subjects were excluded from the hedonic evaluation of essential oils, considering that young adults and middle-aged subjects did not show any anosmia and mild cognitive decline (such as a MoCA score < 26). Non-trained participants evaluated odor dimensions of rosemary and *C. carvi* EOs using a hedonic self-reported Likert scale method [26,50,51]. Before the sensory assessment, each EO was aliquoted at room temperature (23 °C) in 2 mL glass test bottles. EOs were tested for the olfactory analyses in a randomized order and without any dilution. The participants estimated the odor pleasantness, intensity, and familiarity of the two EOs with individual sensory descriptions. The odor pleasantness, intensity, and familiarity of the EOs were estimated by means of a 7-point Likert-type scale, which ranged from 0/not at all to 6 (0 = very unpleasant and 6 = very pleasant; 0 = not intense at all and 6 = very intense; 0 = not familiar at all and 6 = very familiar). The value of 3 was considered a neutral point [26,50,51].

### 2.7. Statistical Analysis

Initially, a sample size calculation was used to estimate the minimum required number of participants for this study using G*Power 3.1. At first, sample size calculation was performed to assess the required minimum number of subjects to be enrolled in the study. Based on previous studies [26,50,51] using similar protocols, a number of about 400 participants was considered adequate to detect the investigated differences. In fact, a power calculation, based on similar studies and considering a critical effect size f = 0.25 (medium effect), with 85–90% power and a 5% significance level in a standard one-way ANOVA, a power calculation considering a critical effect size d = 0.5 (medium effect), with 85–90% power, and a two-tailed 5% significance level in an unpaired t test, suggested a required minimal number of around 59 participants for each group.

The Shapiro-Wilk test was conducted to assess the normality of the data distribution. The test indicated significant deviation from normality only for age, height, weight, and BMI. Consequently, statistical differences between the age groups for the demographic data (height, weight, and BMI) were assessed using the Mann-Whitney U test. One-way between-subjects ANOVAs and post hoc analyses using Bonferroni’s multiple pairwise comparison test were carried out to evaluate statistical differences in olfactory, gustatory, and cognitive functions between the three age ranges: 18–29 years (young adults), 30–59 years (middle-aged), and ≥60 years (elderly). Instead, significant differences in the olfactory ratings between rosemary and caraway EOs were determined by one-way repeated-measures analyses of variance (ANOVA) adjusted with the Bonferroni multiple pairwise comparison tests. For each statistical model, the F-ratio, explaining the source of variance, the degrees of freedom representing the number of groups or factors being compared minus 1, and the degrees of freedom representing the total sample size minus the number of groups, were reported. Partial eta squared (η^2^) estimates the effect size, which provides a measure of the size or the magnitude of the effect.

Statistical differences between percentages of subjects with hyposmia, anosmia, hypogeusia, and ageusia were calculated by Fisher’s exact test. Bivariate correlations between OT, OD, OI, and each subscore of the MoCA scale, such as attention, memory, language, and visuospatial/executive, were calculated by the Pearson’s coefficient (*r*). Finally, a multivariate linear regression analysis was performed to examine the potential contributions of olfactory and gustatory function to attention, memory, language, and visuospatial/executive dysfunction. Adjusted R-squared (R^2^) indicates the overall explanatory power of the model. Statistical analyses were performed by means of SPSS software version 25 for Windows (IBM, Armonk, NY, USA). All data are presented as mean ± standard deviation. The significance level was set at *p* < 0.05 to assess the potential statistical differences.

## 3. Results

### 3.1. EO Chemical Composition

The volatile compounds of *R. officinalis* (EO 1) and *C. carvi* (EO 2) EOs were analyzed by GC/MS technique, and their chemical composition (expressed as % peak area) is reported in Table 1.

GC-MS analysis allowed us to identify 28 compounds in EO 1. Among them, α-pinene was found to be the major component, accounting for 31.8%, followed by 1,8-cineole (13.1%), verbenone (8.4%), (E)-caryophyllene (7.1%), borneol (4.9%), camphene (4.8%), and limonene (4.2%). Other components, with relatively small amounts, were β-pinene (2.5%), terpinolene (2.0%), and bornyl acetate (2.0%). Carvone (53.0%) and limonene (46.4%) represented the most abundant volatile compounds found in EO 2, followed by small amounts of myrcene (0.5%) and (E)-caryophyllene (0.2%).

### 3.2. Demographics of the General Population

Table 2 reports the mean values ± standard deviation (SD) for weight, height, and BMI in the three age ranges. No significant differences were observed among the three age groups for the demographic parameters such as weight, height, and BMI.

### 3.3. Age-Related Sensory Changes

**Figure 1:**
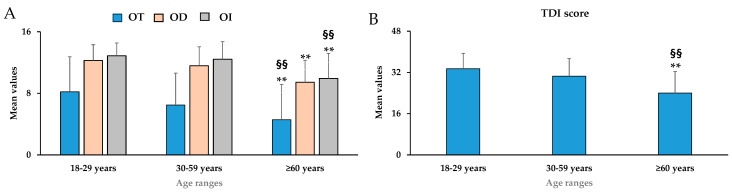
Mean values ± standard deviation (SD) for odor threshold (OT), odor discrimination (OD), odor identification (OI) (**A**), and the global olfactory function as their sum (TDI score) (**B**) in young adults (18–29 years, *n* = 178), middle-aged (30–59 years, *n* = 137), and elderly (≥60 years, *n* = 87). ** = *p* < 0.01 between subjects with 18–29 years compared to those with ≥ 60 years; ^§§^ = *p* < 0.01 between 30–59 years compared to ≥60 years. Statistical differences were calculated by one-way ANOVA followed by Bonferroni’s post hoc test.

Figure 1A,B showed mean values ± standard deviation (SD) for OT, OD, OI, and their sum (TDI score) in young adults (18–29 years), middle-aged (30–59 years), and elderly (≥60 years). The elderly group showed a significant decline in the mean scores of OT [F_(2,399)_ = 20.51, *p* < 0.01, partial η^2^ = 0.093], OD [F_(2,399)_ = 42.08, *p* < 0.01, partial η^2^ = 0.174], OI [F_(2,399)_ = 49.99, *p* < 0.01, partial η^2^ = 0.200], and TDI score [F_(2,399)_ = 55.69, *p* < 0.01, partial η^2^ = 0.218] (Figure 1A,B). Additionally, significant differences (*p* < 0.01) were observed in the OT, OI, and TDI mean scores between the 30–59 years group (middle-aged) and the ≥60 years group (elderly).

Considering the TDI mean score, a significant increase (*p* < 0.01) in the percentage of anosmia was observed among young adults (18–29 years), middle-aged individuals (30–59 years), and the elderly (≥60 years) (Table 3). Anosmia prevalence increased significantly with age, reaching 20.7% in the ≥60 group (*p* < 0.01). Instead, a significant increase (*p* < 0.01) in the percentage of hypogeusia was observed only between young adults (18–29 years) and the elderly (≥60 years) group. Gustatory function decreased in relation to age differently from olfactory function; low percentages of ageusia were shown in the three age groups (Table 3).

**Figure 2:**
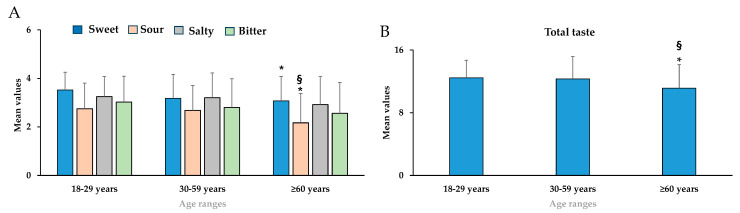
Mean values ± standard deviation for sweet, sour, salty, bitter (**A**), and global taste perception (**B**) in young adults (18–29 years, *n* = 178), middle-aged (30–59 years, *n* = 137), and elderly (≥60 years, *n* = 87). * = *p* < 0.05 between subjects with 18–29 years compared to those with ≥60 years; ^§^ = *p* < 0.05 between 30–59 years compared to ≥60 years. Statistical differences were calculated by one-way ANOVA followed by Bonferroni’s post hoc test.

Figure 2A,B showed mean values ± standard deviation for sweet, sour, salty, bitter (A), and global taste perception in young adults (18–29 years), middle-aged (30–59 years), and elderly (≥60 years).

Gustatory function decreased in relation to age differently from olfaction, since only mean values of sweet [F_(2,399)_ = 9.56, *p* < 0.05, partial η^2^ = 0.046] and sour [F_(2,399)_ = 8.93, *p* < 0.05, partial η^2^ = 0.043] taste perceptions significantly decreased in elderly participants (≥60 years) compared to the young adults’ group (18–29 years) (Figure 2).

### 3.4. Age-Related Cognitive Changes

**Figure 3:**
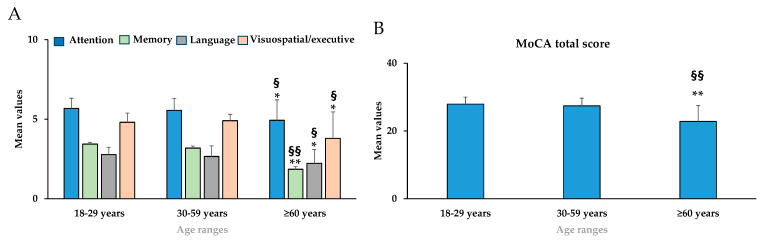
Mean values ± standard deviation for attention, memory, language, visuospatial/executive (**A**) subscores of cognitive abilities and cognitive total score (MoCA) (**B**) obtained using Montreal cognitive abilities (MoCA) scale in young adults (18–29 years, *n* = 178), middle-aged (30–59 years, *n* = 137), and elderly (≥60 years, *n* = 87). * = *p* < 0.05 between 18–29 years compared to ≥ 60 years; ** = *p* < 0.01 between subjects with 18–29 years compared to those with ≥ 60 years; ^§^ = *p* < 0.05 and ^§§^ = *p* < 0.01 between 30–59 years compared to ≥60 years. Statistical differences were calculated by one-way ANOVA followed by Bonferroni’s post hoc test.

Figure 3A,B showed mean values ± standard deviation for attention, memory, language, visuospatial/executive subscores of cognitive abilities, and the cognitive total score (MoCA) in young adults (18–29 years), middle-aged (30–59 years), and elderly (≥60 years) groups. In the cognitive abilities, the mean values of subscores of attention [F_(2,399)_ = 22.53, *p* < 0.01, partial η^2^ = 0.101], memory [F_(2,399)_ = 33.06, *p* < 0.01, partial η^2^ = 0.142], language [F_(2,399)_ = 22.07, *p* < 0.05, partial η^2^ = 0.100], and visuospatial/executive [F_(2,399)_ = 44.73, *p* < 0.05, partial η^2^ = 0.183] showed a significant decline in the elderly group (≥60 years) compared to the young adults’ group (18–29 years) (Figure 3A).

Moreover, we observed a significant decrease in the mean values of attention, memory, language, and visuospatial/executive subscores between the middle-aged (30–59 years) and the elderly (≥60 years) group (Figure 3A). Consequently, the mean values of MoCA total score [F_(2,399)_ = 59.85, *p* < 0.01, partial η^2^ = 0.231] decreased significantly in the elderly group (≥60 years) compared to the young adults’ group (18–29 years) and the middle-aged (30–59 years) group (Figure 3B).

### 3.5. Sensory-Cognitive Associations

A negative significant correlation was observed between the age versus OT (*r* = −0.305, *p* < 0.01), OD (*r* = −0.407, *p* < 0.01), OI (*r* = −0.428, *p* < 0.01), sweet (*r* = −0.168, *p* < 0.05), sour (*r* = −0.176, *p* < 0.05), salty (*r* = −0.132, *p* < 0.05), bitter (*r* = −0.126, *p* < 0.05), attention (*r* = −0.320, *p* < 0.01), memory (*r* = −0.389, *p* < 0.01), language (*r* = −0.335, *p* < 0.01), visuospatial/executive (*r* = −0.372, *p* < 0.01), and MoCA total score (*r* = −0.471, *p* < 0.01).

**Figure 4:**
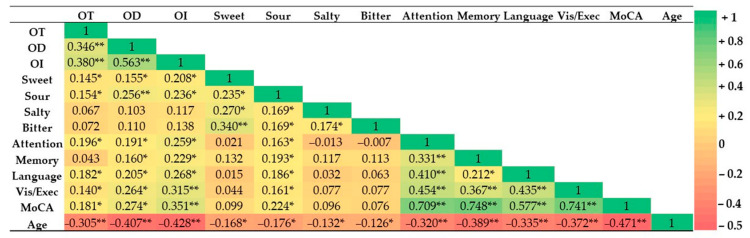
Heatmap of Pearson’s correlations (*r*) for odor threshold (OT), odor discrimination (OD), odor identification (OI), and gustatory perception (sweet, salty, sour, and bitter) versus each subscore of the Montreal Cognitive (MoCA) Scale, such as attention, memory, language, visuospatial/executive, and global cognitive score. ** = *p* < 0.01; * = *p* < 0.05.

In the olfactory function, significant correlations were observed between OI and each subscore of cognitive abilities as attention (*r* = 0.259, *p* < 0.01), memory (*r* = 0.229, *p* < 0.01), language (*r* = 0.268, *p* < 0.01), visuospatial/executive (*r* = 0.315, *p* < 0.01), and also for the global cognitive score (MoCA) (*r* = 0.351, *p* < 0.01). In addition, slightly significant correlations were found between OT (*r* = 0.196, *p* < 0.05; *r* = 0.182, *p* < 0.05; *r* = 0.140, *p* < 0.05, respectively) and OD (*r* = 0.191, *p* < 0.05; *r* = 0.205, *p* < 0.05; *r* = 0.264, *p* < 0.01), versus the attention, language, and visuospatial/executive subscores of cognitive abilities (Figure 4).

Instead, in the gustatory function, significant correlations were found between sour taste perception versus attention (*r* = 0.163, *p* < 0.05), memory (*r* = 0.193, *p* < 0.05), language (*r* = 0.186, *p* < 0.05), and MoCA total score (*r* = 0.224, *p* < 0.01).

### 3.6. Regression Analyses

Finally, to better understand how olfactory and gustatory functions could foster cognitive abilities, an exploratory multiple regression analysis was conducted for attention, memory, language, and the MoCA total score.

Table 4 presents multiple regression analyses using each subscore of cognitive abilities, such as attention, memory, language, and the MoCA total score, as dependent variables.

The OI and sour taste perception were significantly associated with each cognitive subscore, namely attention [F_(7,401)_ = 5.990, *p* < 0.001, adjusted R^2^ = 0.080], memory [F_(7,401)_ = 5.296, *p* < 0.001, adjusted R^2^ = 0.070], language [F_(7,401)_ = 6.364, *p* < 0.001, adjusted R^2^ = 0.086], and global cognitive function (MoCA score) [F_(7,401)_ = 10.056, *p* < 0.001, adjusted R^2^ = 0.137]. The model explained around 7–8% of the variance for attention, memory, and language, while the global cognitive function explained around 15% of the variance.

### 3.7. EOs Hedonic Ratings

Our data showed that OT, OD, OI, sour taste perception, and each subscore of cognitive abilities decrease in relation to age. Consequently, we have abolished the elderly age group and focused our attention on a subpopulation of young adults (18–29 years) and middle-aged (30–65 years) participants (*n* = 59, 39 women and 20 men) to better understand the potential role of some EOs in the modulation of the chemosensory system. Demographic parameters of the subpopulation are indicated in Table 5.

No significant differences were found between the two subgroups for weight, height, and BMI.

**Figure 5:**
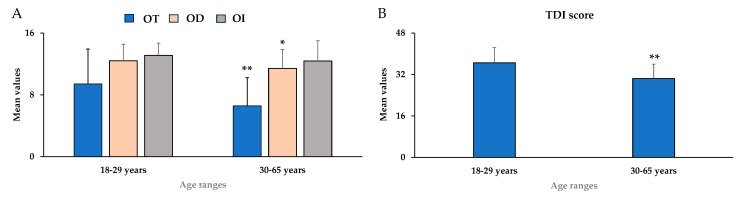
Mean values ± standard deviation for odor threshold (OT), odor discrimination (OD), odor identification (OI) (**A**), and the global olfactory function, their sum (TDI score) (**B**) in young adults (18–29 years, *n* = 40) and middle-aged (30–65 years, *n* = 19). * = *p* < 0.05; ** = *p* < 0.01. Statistical differences were calculated by one-way ANOVA followed by Bonferroni’s post hoc test.

Figure 5A,B showed mean values ± standard deviation for OT, OD, OI, and the global olfactory function TDI score in young adults (18–29 years) and middle-aged (30–65 years).

The subpopulation exhibited substantial differences between the two age ranges for the mean values of OT [F_(1,56)_ = 12.64, *p* < 0.01, partial η^2^ = 0.184], OD [F_(1,56)_ = 5.42, *p* < 0.05, partial η^2^ = 0.088], and TDI score [F_(1,56)_ = 14.351, *p* < 0.01, partial η^2^ = 0.204] (Figure 5A,B), which were consistent with the trends observed in the general population (Figure 5A,B). Instead, no significant differences were observed for OI mean values.

Carvone and limonene, the primary components of caraway in the chemical composition analysis, were correctly identified by approximately 70% of the participants in the discrimination task.

Within the subpopulation, we intentionally excluded participants with anosmia. However, mild hyposmia was observed in 10% (*n* = 4) and in 42% of young adults (18–29 years, *n* = 40) and the middle-aged group, respectively.

Considering gustatory function, as reported in the general population, we did not find significant differences in the perception of different taste modalities between the 18–29 and 30–65 age groups. Moreover, only 16% (*n* =8) of participants in the age range 30–65 years showed mild hypogeusia.

As regards cognitive performance, in our subpopulation, we did not observe any significant difference, as reported in the general population, for each subscore of cognitive abilities (attention, memory, language, and visuospatial/executive function) between the 18–29 and 30–65 age groups. Consequently, in this subpopulation, we did not observe any decrease in cognitive performance between the age groups.

**Figure 6:**
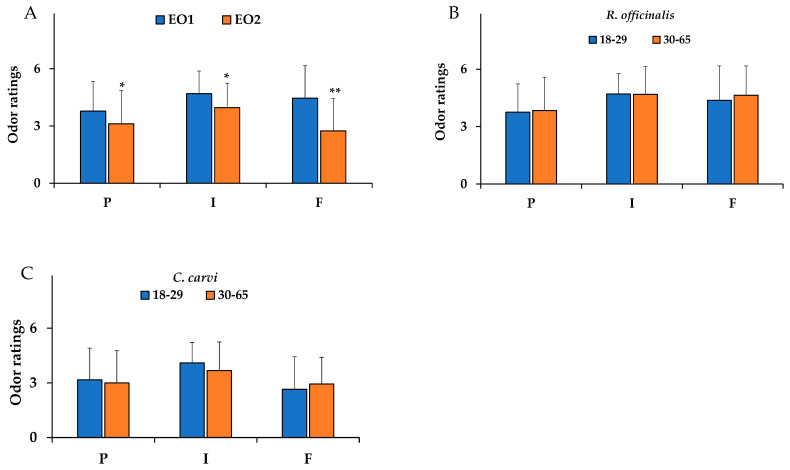
Mean values ± standard deviation for odor ratings of pleasantness (P), intensity (I), and familiarity (F) dimensions for *R. officinalis* and *C. carvi* essential oils (EO 1 and EO 2, respectively), considering the total subpopulation (**A**), and divided into two age ranges: 18–29 years and 30–65 years (**B**,**C**). Data are indicated as mean values ± standard deviation. * = *p* < 0.05; ** = *p* < 0.05 between *R. officinalis* (EO 1) and *C. carvi* (EO 2) EOs. Statistical differences were calculated by one-way repeated-measures analyses of variance (ANOVA) adjusted with the Bonferroni multiple pairwise comparison tests.

Figure 6A shows mean values ± standard deviation of the odor ratings for pleasantness (P), intensity (I), and familiarity (F) dimensions for the two EOs, *R. officinalis* (EO 1) and *C. carvi* (EO 2), in the subpopulation.

The main effect of the EO type within-subjects factor was statistically significant [F_(1,112)_ = 10.192, *p* < 0.05, partial η^2^ = 0.154]. In the pairwise analysis, rosemary (EO 1), which contains α-pinene and 1,8-cineole as the major components, was perceived as more pleasant, more intense, and more familiar than the caraway (EO 2) (Figure 6A). The caraway EO 2, even though it contains carvone and limonene, two chemical substances that are easily distinguishable by 70% of participants, is considered less pleasant, less intense, and less familiar than rosemary.

The main effect of the age group within-subjects factor was not statistically significant (*p* > 0.05). Interestingly, in the pairwise analysis, no significant differences were observed in the odor ratings of rosemary (Figure 6B) and caraway EOs (Figure 6C) with respect to the odor pleasantness, intensity, and familiarity dimensions between young adults (18–29 years) and middle-aged (30–65 years) groups. The two age groups perceived these EOs without any significant decrease in odor ratings in relation to aging.

Participants also provided subjective descriptions of the two EOs, and the results are shown in Table 6. The sole discernible distinction was a greater propensity to describe the EOs with greater linguistic precision in the 30–65 age group.

Regarding EO 1 (rosemary), some participants in both age groups recognized the exact EO, probably due to the high concentration of the main components α-pinene and 1,8-cineole.

EO 2 (caraway), on the other hand, is characterized by an odor that is little known among the population of any age group. Therefore, it was not identified in any age group, but several sensory descriptions with possible similarities were provided, such as mint, eucalyptus, balsamic plant, and artichoke.

## 4. Discussion

### 4.1. Age-Related Changes in Olfactory, Gustatory Functions, and Cognitive Abilities

Our study initially aimed to evaluate the age-related changes in olfactory (OT, OD, and OI), gustatory (sweet, sour, salty, and bitter taste perceptions), and cognitive functions (focusing on attention, memory, language, and visuospatial/executive function), and their potential correlations with aging. In a population of 402 participants, our results indicated that OT, OD, OI, attention, memory, language, and visuospatial/executive abilities decreased in relation to age.

Aging is characterized by a decline in olfactory, gustatory, and cognitive functions. In particular, OT, OD, OI, attention, memory, language, and visuospatial/executive functions showed a significant decrease around 60 years, as previously reported [3,14,52]. Age-related decline in olfactory function may negatively affect daily life in older adults, causing changes in eating habits and many adverse effects, including decreased food enjoyment, altered nutritional choices, and a higher risk of malnutrition. These negative effects linked to age-related olfactory dysfunction may lead to chronic diseases such as dyslipidemia, diabetes mellitus, and hypertension. Olfactory age-related dysfunction could be explained by the reduced receptor numbers in the nasal epithelium, brain atrophy, and decreased olfactory pathways in the olfactory bulb, piriform cortex, and amygdala [14,53].

Our results showed that gustatory function declines with age more slowly than olfaction, as only sweet and sour taste perceptions significantly decreased in elderly participants (≥60 years) compared to the young adult (18–29 years) group. In contrast, salty and bitter taste perceptions did not decline with age. Our data, which aligns with a previous study, indicated that the decline in taste perception with age varies across different taste modalities [54]. Our findings differed from those reported by Alia and colleagues [54], who found that only salty and bitter taste perceptions decreased in elderly subjects. This discrepancy could be due to differences in the inclusion criteria, particularly considering variations in food consumption and eating behavior among the elderly participants. The decrease in gustatory function could be related both to an impairment in the peripheral gustatory system, such as taste buds, and in the central gustatory system, such as in the cerebellum [55]. The age-related olfactory and gustatory deficits are considered mechanisms of the normal aging process [14,56,57].

Olfactory and gustatory deficits are often associated with cognitive dysfunction, such as a reduction in attention, memory, language, and executive function. Our data indicated that OI could be considered a potential early indicator for attention, memory, language, and visuospatial/executive dysfunction. These results are consistent with previous studies [58,59], suggesting an association between olfactory function and attention, memory, and visuospatial/executive functions. Odor identification and cognitive abilities showed interconnected pathways involving the orbitofrontal cortex, hippocampus, and amygdala [60]. Consequently, the combination of olfactory tests and cognitive abilities scales may contribute to detecting subjects with a high risk of cognitive decline. Elderly subjects with olfactory and cognitive deficits showed a high risk of neurodegenerative diseases, including Alzheimer’s disease and dementia [61]. Considering associations between gustatory and cognitive functions, a low correlation was found between sour taste perception and cognitive abilities, while Makizako and colleagues [58] did not observe any association between taste and each cognitive subscore.

### 4.2. Effects of R. officinalis and C. carvi EOs on the Chemosensory System in Relation to Age

In the second part of the study, we demonstrated that *R. officinalis* and *C. carvi* EOs improved chemosensory perception in relation to age using olfactory procedures in a subpopulation of young adults and middle-aged participants. The use of natural EOs is a strategy that can promote healthy aging and has recently gained attention. Natural EOs are often used for their biological activities to improve cognitive function and patients’ quality of life [21,24,25,27,28,29]. Moreover, it is well known that EO inhalation plays a role in the improvement of olfactory perception [26,62,63]. EOs enhance the regeneration of olfactory neurons and facilitate the recovery of olfactory sensory function [63]. The study of olfactory function has increased in recent years, and the olfactory system can be a marker of neurodegeneration in aging [63].

In our study, odor pleasantness, intensity, and familiarity of rosemary and caraway EOs were assessed in young adults and middle-aged participants. According to the general population, the subpopulation showed significant differences in olfactory function in relation to age. Our data indicated that rosemary EO 1 was perceived without any significant decrease in odor pleasantness, intensity, and familiarity ratings in relation to age. Similarly, no significant differences emerged in the olfactory perception (odor ratings of pleasantness, intensity, and familiarity) of caraway EO 2 between young adults and middle-aged participants. Our results highlighted the possible role of both EOs in the modulation of olfactory perception.

Aromatherapy-based olfactory training involves the use of natural plant EOs to influence the olfactory system by leveraging their volatile components [64,65]. The inhalation of EOs is considered an emerging non-pharmacological intervention to ameliorate olfactory dysfunction for their ability to promote olfactory nerve plasticity, enhance the regeneration of olfactory neurons, and modify the plasticity of central olfactory pathways [63]. EOs contain volatile compounds, which are lipophilic in nature that may cross the blood–brain barrier with direct bioactivity in the central nervous system, as previously indicated [21,24].

In addition to antioxidant and anti-inflammatory effects, previous studies have indicated that rosemary EO exerts important effects on central nervous systems [21,32,33]. Inhalation of rosemary EO has been demonstrated to improve memory, attention, cognitive function, and mood [21,66]. A previous study found that rosemary EO produced a significant improvement in memory abilities and cognitive performance [66]. Rosemary EO is amply used in aromatherapy to improve the chemosensory system and the cognitive performance in elderly subjects and in Alzheimer’s disease [67].

According to the literature, EO 1 was characterized by α-pinene (31.8%) and 1,8-cineole (13.1%) as the major volatile components. 1,8-Cineole activates neuronal responses in the lateral/ventral part of the olfactory epithelium and olfactory bulb [63,68]. In addition, 1,8-cineole is described as a trigeminal stimulus with a cooling effect [36]. The trigeminal nerve mediates nociception in the nasal cavity [11].

The anti-inflammatory and antioxidant properties of caraway EO have been previously reported [37,38]. A recent study evidenced the bioactivity of caraway EO on the central nervous system in the improvement of memory abilities [69]. The neuroprotective effect of caraway EO has been explained by its accumulation in the hippocampus and in the modulation of the AChE activity enzyme [69]. Limonene (46.4%) and carvone (53.0%) emerged as the major volatile components of EO 2 [38]. Limonene, like 1,8-cineole, showed neuronal responses in the lateral/ventral part of the olfactory epithelium and olfactory bulb, while carvone showed such responses in the dorsal region of the olfactory epithelium and olfactory bulb [63,68]. Also, carvone and limonene are described as trigeminal stimuli with a cooling effect [36]. Carvone can cross the blood–brain barrier and may be accumulated in the hippocampus [69]. Both carvone and limonene have been reported to improve cognition in neurodegenerative diseases [70,71].

The comparison between rosemary and caraway EOs indicated that subjects perceived rosemary EO as more pleasant, more intense, and more familiar than caraway EO.

These results could be explained by considering their chemical composition and the different odorant receptor distribution in the olfactory system. 1,8-Cineole, carvone, and limonene are terpene compounds that could generate different effects on the olfactory system in relation to diverse types of olfactory sensory neurons activated [63].

The similar perception of both EOs’ odor dimensions in middle-aged participants compared with young adults could be partially explained by the potential trigeminal activation due to their main volatiles.

Given the cross-sectional design, causal relationships cannot be inferred. The limitations of this study include the low number of participants for EO sensory assessment, the cross-sectional design, and the absence of a longitudinal design to better understand the specific role of rosemary and caraway on cognitive abilities. In addition, odor ratings of pleasantness, intensity, and familiarity dimensions for rosemary and caraway EOs could be influenced by cultural habits.

## 5. Conclusions

Aging is characterized by a decrease in olfactory, attentional, memory, language, and visuospatial/executive abilities. Olfactory function was significantly associated with attention, memory, language, and visuospatial/executive dysfunction. Gustatory function decreased in relation to age differently from olfaction, since only mean values of sweet and sour taste perceptions significantly decreased in elderly participants (≥60 years) compared to the young adults’ group (18–29 years). Olfactory testing combined with cognitive assessment can be an excellent tool for the early diagnosis of mild cognitive impairment. Our data indicated that rosemary and caraway EOs were perceived without any significant decrease in odor pleasantness, intensity, and familiarity ratings in relation to aging. The preserved perception of rosemary and caraway EOs across age groups suggests their potential suitability for future interventional studies. Further studies will be needed to assess the potential role of these EOs in improving cognitive abilities over the long term.

## Figures and Tables

**Table 1:** Profile, retention index (RI_EXP_), retention index from the literature (RI_LIT_), formula, class, and % amount (semi-quantitative analysis by peak area normalization) of the main identified volatile compounds of EO 1 and EO 2 evaluated by GC-MS.

					EO 1	EO 2
Compound	^1^ RI_EXP_	RI_LIT_	Formula	Class	Amount (%)	
Tricyclene	926	921	C_10_H_16_	MH	0.2	-
α-Thujene	927	924	C_10_H_16_	MH	0.2	-
α-Pinene	935	932	C_10_H_16_	MH	31.8	-
Camphene	951	946	C_10_H_16_	MH	4.8	-
Thuja-2,4(10)-diene	957	953	C_10_H_14_	MH	1.1	-
β-Pinene	980	974	C_10_H_16_	MH	2.5	-
Myrcene	989	988	C_10_H_16_	MH	1.6	0.5
α-Phellandrene	1007	1002	C_10_H_16_	MH	0.6	-
iso-Sylvestrene	1012	1007	C_10_H_16_	MH	1.5	-
α-Terpinene	1017	1014	C_10_H_16_	MH	0.8	-
para-Cymene	1024	1020	C_10_H_14_	MH	1.4	-
Limonene	1031	1024	C_10_H_16_	MH	4.2	46.4
1,8-Cineole	1034	1026	C_10_H_18_O	MO	13.1	-
γ-Terpinene	1057	1054	C_10_H_16_	MH	1.3	-
Terpinolene	1083	1086	C_10_H_16_	MH	2.0	-
Linalool	1099	1095	C_10_H_18_O	MO	1.6	-
Chrysanthenone	1126	1124	C_10_H_14_O	MO	1.3	-
Camphor	1147	1141	C_10_H_16_O	MO	1.8	-
Borneol	1167	1165	C_10_H_18_O	MO	4.9	-
cis-Pinocamphone	1175	1172	C_10_H_16_O	MO	0.3	-
Terpin-4-ol	1178	1174	C_10_H_18_O	MO	1.1	-
α-terpineol	1191	1186	C_10_H_18_O	MO	1.9	-
Myrtenol	1196	1194	C_10_H_16_O	MO	0.7	-
trans-Dihydrocarvone	1203	1200	C_10_H_16_O	MO	0.5	-
Verbenone	1209	1204	C_10_H_14_O	MO	8.4	-
Carvone	1231	1239	C_10_H_12_O	MO	-	53.0
Bornyl acetate	1286	1287	C_12_H_20_O_2_	MO	2.0	-
*(E)*-Caryophyllene	1412	1417	C_15_H_24_	SH	7.1	0.2
α-Humulene	1448	1452	C_15_H_24_	SH	1.0	-
Unidentified peaks					-	-

Legend: ^1^ RI_EXP_, retention index determined on a DB-5MS UI fused silica column relative to a series of *n*-alkanes [39,40]; MH = monoterpene hydrocarbons; MO = oxygen-containing monoterpenes; SH = sesquiterpene hydrocarbons.

**Table 2:** Demographic information expressed as mean values ± standard deviation (SD) in the following age ranges: 18–29 years (young adults), 30–59 years (middle-aged), and ≥60 years (elderly).

Parameters	18–29 Years (*n* = 178)	30–59 Years (*n* = 137)	≥60 Years (*n* = 87)
Sex	115 W/63 M	63 W/74 M	48 W/39 M
Weight (kg)	62.9 ± 13.9	68.4 ± 13.2	69.8 ± 14.4
Height (m)	1.7 ± 0.1	1.67 ± 0.1	1.6 ± 0.1
BMI	22.9 ± 4.1	25.9 ± 7.4	25.7 ± 4.1

Legend: BMI = body mass index; M = men; W = women. Significant differences among the three age ranges were assessed using the Mann–Whitney U test.

**Table 3:** Percentages of olfactory and gustatory deficits in the following age ranges: 18–29 years (young adults), 30–59 years (middle-aged), and ≥60 years (elderly).

Parameters	18–29 Years (*n* = 178)	30–59 Years (*n* = 137)	≥60 Years (*n* = 87)
Anosmia	1.1%	2.2%	20.7% **
Hyposmia	24.3%	40.1%	54% **
Ageusia	0	1.5%	2.3%
Hypogeusia	11.8%	15.3%	25.3% **

Legend: Anosmia = TDI scores ≤ 16; hyposmia = TDI scores between 16.25 and 30.5; ageusia = patients without any sensation of the highest concentrations for each taste modality; hypogeusia = Total Taste scores < 9. ** = *p* < 0.01 Fisher’s exact test.

**Table 4:** Multiple regression analyses using gustatory and olfactory parameters as predictors for cognitive abilities.

Predictors	B	SD Error	β	t	Significance (*p* Value)
(a) Attention as a dependent variable					
OT	0.021	0.010	0.106	2.002	0.046
OD	0.011	0.020	0.031	0.516	0.606
OI	0.068	0.021	0.194	3.212	**0.001**
Sweet	−0.039	0.053	−0.039	−0.734	0.463
Sour	0.096	0.042	0.118	2.295	**0.022**
Salty	−0.042	0.044	−0.048	−0.955	0.340
Bitter	−0.033	0.040	−0.043	−0.838	0.402
(b) Memory as a dependent variable					
OT	−0.025	0.019	−0.071	−1.333	0.183
OD	0.018	0.037	0.029	0.477	0.634
OI	0.121	0.039	0.190	3.123	**0.002**
Sweet	0.070	0.097	0.039	0.720	0.472
Sour	0.186	0.076	0.125	2.435	**0.015**
Salty	0.092	0.081	0.057	1.129	0.259
Bitter	0.061	0.072	0.044	0.849	0.396
(c) Language as a dependent variable					
OT	0.012	0.008	0.083	1.588	0.113
OD	−0.011	0.015	0.042	0.706	0.481
OI	0.051	0.016	0.196	3.253	**0.001**
Sweet	−0.063	0.040	−0.085	−1.593	0.112
Sour	0.080	0.031	0.132	2.583	**0.010**
Salty	−0.004	0.033	−0.006	−0.119	0.905
Bitter	0.019	0.030	0.033	0.636	0.525
(d) MoCA as a dependent variable					
OT	0.023	0.036	0.033	0.640	0.522
OD	0.101	0.072	0.080	1.392	0.165
OI	0.334	0.075	0.261	4.452	**0.001**
Sweet	−0.053	0.188	−0.015	−0.281	0.779
Sour	0.396	0.148	0.133	2.680	**0.008**
Salty	0.114	0.157	0.035	0.727	0.468
Bitter	0.014	0.140	0.005	0.101	0.919

Legend: OT = Odor threshold; OD = Odor discrimination; OI = Odor identification. B = unstandardized coefficient for each predictor variable; β = standardized coefficient, which gives a measure of the variable contribution; SD = standard; t = t-values, which indicate whether the predictor’s regression coefficient is significant. Bold indicates the significance level.

**Table 5:** Demographic information of the subpopulation expressed as mean values ± standard deviation in the following age ranges: 18–29 years (young adults) and 30–65 years (middle-aged).

Parameters	18–29 Years (*n* = 40)	30–65 Years (*n* = 19)
Sex	28 W/12 M	11 W/8 M
Weight (kg)	61.2 ± 11.8	65.5 ± 9.5
Height (m)	1.6 ± 0.1	1.7 ± 0.1
BMI	22.9 ± 4.1	23.7 ± 2.4

Legend: BMI = body mass index. M = men; W = women; Significant differences between the two age ranges were assessed by one-way ANOVAs.

**Table 6:** Odor perceived description for EO 1 (*R. officinalis*) and EO 2 (*C. carvi*) measured in young adults (18–29 years) and middle-aged (30–65 years) groups with main constituents and chemical sensation.

EO	EOs Perceived Odor	Main Constituents	Chemical Sensation
*R. officinalis*	*18–29 years:* Rosemary, lavender, and turpentine *30–65 years:* Citrus fruit, natural essence, spice, rosemary, bergamot, saffron, menthol, eucalyptus, and plant bark	α-Pinene	
		1,8-Cineole	Trigeminal [25]
	
*C. carvi*	*18–29 years:* Vanilla, liquorice, herbs, anise, balsamic plants, and almond *30–65 years:* Mint, unpleasant, aromatic, menthol, eucalyptus, balsamic plants, and artichoke	Carvone	Trigeminal [25]
		Limonene	Trigeminal [25]
	

## Data Availability

The datasets generated and analyzed during the current study, due to ethical restrictions, are available from the corresponding author on reasonable request.

## Abbreviations

The following abbreviations are used in this manuscript:

AChE	Acetylcholinesterase
EO	Essential Oil
EO 1	*Rosmarinus officinalis* Essential Oil
EO 2	*Carum carvi* Essential Oil
MoCA	Montreal Cognitive Assessment
OT	Odor Threshold
OD	Odor Discrimination
OI	Odor Identification
ROS	Reactive Oxygen Species
TDI	Threshold + Discrimination + Identification
